# Genome-Wide Identification of miRNAs and Their Targets Involved in the Developing Internodes under Maize Ears by Responding to Hormone Signaling

**DOI:** 10.1371/journal.pone.0164026

**Published:** 2016-10-03

**Authors:** Zhan Zhao, Yadong Xue, Huili Yang, Huimin Li, Gaoyang Sun, Xiaofeng Zhao, Dong Ding, Jihua Tang

**Affiliations:** 1 National Key Laboratory of Wheat and Maize Crop Science, Collaborative Innovation Center of Henan Grain Crops, College of Agronomy, Henan Agricultural University, Zhengzhou, 450002, Henan, China; 2 Hubei Collaborative Innovation Center for Grain Industry, Yangtze University, Jingzhou, 434023, Hubei, China; University of Balochistan, PAKISTAN

## Abstract

Internode length is one of the decisive factors affecting plant height (PH) and ear height (EH), which are closely associated with the lodging resistance, biomass and grain yield of maize. miRNAs, currently recognized as important transcriptional/ post-transcriptional regulators, play an essential role in plant growth and development. However, their roles in developing internodes under maize ears remain unclear. To identify the roles of miRNAs and their targets in the development of internodes under maize ears, six miRNA and two degradome libraries were constructed using the 7^th^, 8^th^ and 9^th^ internodes of two inbred lines, ‘Xun928’ and ‘Xun9058’, which had significantly different internode lengths. A total of 45 and 54 miRNAs showed significant changes for each pairwise comparison among the 7^th^, 8^th^ and 9^th^ internodes of ‘Xun9058’ and ‘Xun928’, respectively. The expression of 31 miRNAs showed significant changes were common to the corresponding comparison groups of the 7^th^, 8^th^ and 9^th^ internodes of ‘Xun9058’ and ‘Xun928’. For the corresponding internodes of ‘Xun9058’ and ‘Xun928’, compared with the expression of miRNAs in the 7^th^, 8^th^ and 9^th^ internodes of ‘Xun928’, the numbers of up-regulated and down-regulated miRNAs were 11 and 36 in the 7^th^ internode, 9 and 45 in the 8^th^ internode, and 9 and 25 in the 9^th^ internode of ‘Xun9058’, respectively. Moreover, 10 miRNA families containing 45 members showed significant changes at least in two internodes of ‘Xun928’ by comparing with the corresponding internodes of ‘Xun9058’. Based on the sequencing data, 20 miRNAs related to hormone signaling among the candidates, belonging to five conserved miRNA families, were selected for expression profiling using quantitative reverse-transcription polymerase chain reaction (qRT-PCR). The five miRNA families, zma-miR160, zma-miR167, zma-miR164, zma-miR169 and zma-miR393, targeted the genes encoding auxin response factor, N-acetylcysteine domain containing protein, nuclear transcription factor Y and auxin signaling F-BOX 2 through degradome sequencing. The miRNAs might regulate their targets to respond to hormone signaling, thereby regulating the internode elongation and development under maize ear. These results provide valuable reference for understanding the possible regulation mechanism of the ILs under the ear.

## Introduction

Lodging has become a serious problem in cereal crop production because of the decrease in yield and quality owing to the reduced photosynthesis in the canopy, damaged vascular bundles in bent or broken stems and the effects on mechanical harvesting [1,2]. In maize, lodging may cause a great loss in the annual yield [3]. Several abiotic factors can cause maize lodging in the field, such as heavy rain and high wind. In addition to abiotic factors, some traits related to the prevention of lodging in maize have been studied, such as plant (PH) and ear (EH) height [4,5]. PH and EH are two important agronomic traits in crop breeding programs that are closely associated with biomass, lodging resistance and grain yield [6–8], and are constructed by two components: internode number (IN) and internode length (IL). However, the IN and IL for inbred lines and hybrids can be significantly different.

In maize, some key genes regulate the PH and EH through hormone metabolism and transport or by affecting cell division and/or cell elongation [8, 9]. The genes encoding enzymes (the wild type products) involved in gibberellin (GA) biosynthesis play important roles in increasing PH [10–12]. In addition to these genes, the *brachytic* mutants are the only class that has a short stature owing to a shortening of the IL of lower stalk without having a corresponding reduction in the IN or the size of other organs, and the *Br2* gene encodes a protein involved in auxin polar transport [13]. Additionally, the *dil1* gene annotated as *Activating Protein 2* transcription factor-like is involved in stalk and leaf development in maize [9].

miRNAs are endogenous small RNAs, 20–24 nucleotide in length, which play crucial post-transcriptional regulatory roles in plants [14–16]. miRNAs have important functions in plants and animals and act in guiding mRNA degradation or translational repression in post-transcriptional gene silencing [16,17]. Furthermore, most plant miRNAs contain sequences that are highly complementary to their target binding sites and more than half of the known miRNA-target genes encode TFs in Arabidopsis [18]. The TFs, such as auxin response factor (ARF), N-acetylcysteine domain containing protein (NAC), nuclear transcription factor Y (NF-Y) and auxin signaling F-BOX 2 (AFB2), targeted by miRNAs are reported to respond to hormone stimuli [18–23]. According to Baloch et al. [24], bioinformatic tools have been used widely to identify conserved miRNAs and predict the new miRNAs. During recent decades, with the rapid development of sequencing technology, a growing body of research has demonstrated that miRNAs take part in plant growth and development, including organ identity [25,26], leaf morphology [27,28] and abiotic stress responses [26,29–31]. Next-generation sequencing techniques have revolutionized the miRNA identification methods in model plants, such as Arabidopsis [32] and rice [33,34]. Deep sequencing of small RNAs associated with degradome has become an effective way to identify the roles of miRNAs.

Maize hybrids and inbred lines always have 18–20 internodes, and the basal 5 or 6 internodes do not elongate and remain underground [35]. In the field, stems bent or broken at the aboveground basal internodes in hybrid or inbred lines usually result in lodging, which decreases maize grain yield [36]. Thus the IL and IN under the ear mainly decide the EH and affect the lodging-resistant ability of hybrid or inbred lines. In the previous study, several genes for EH and PH, such as *br2* and *d2003*, were cloned [8,13], and Tang et al. [37] reported that the IL was the main contributor to EH and PH in a maize recombinant inbred line population. In this study, the two inbred lines,‘Xun928’ and ‘Xun9058’,which are the parents of the elite hybrid Xundan20 that was the second most cultivated hybrid in China from 2010 to 2013, were used as the materials. The hybrid with a high EH undergoes lodging in the field easily, and its two parents, ‘Xun928’ and ‘Xun9058’, had significantly different internode lengths from the 7^th^ internode to the 9^th^ internode under the ear. A whole-genome-wide identification of miRNAs was performed based on deep-sequencing technology for detecting the miRNAs related to developing internodes under maize ear. The main goals of this work were to investigate the roles of miRNAs involved in the developing internodes under maize ear and to analyze the regulatory between miRNAs and their target genes related to internode development.

## Methods

### Plant Materials and RNA Isolation

The two inbred lines, ‘Xun928’ and ‘Xun9058’, which were the parents of the elite hybrid Xundan20 that was the second most cultivated hybrid in China from 2010 to 2013, were used as the materials in this study. The hybrid Xundan20 has a high ear height (130-135cm), and undergoes lodging in the field easily. The inbred line ‘Xun9058’ belongs to the Reid germplasm, and ‘Xun928’ is derived from Chinese local germplasm Tangsipingtou. Both of ‘Xun9058’ and ‘Xun928’ were gifts from Hebi Academy of Agricultural Sciences in China. They had different IL and IN under their ears. Field trials were conducted at the farms of Henan Agricultural University (Zhengzhou, China; E113°42′, N34°48′). The inbred lines ‘Xun9058’ and ‘Xun928’ were grown in a random design with three replications at the site. Plots consisted of single row, 0.8 m apart and 4 m long, and were over-planted and later thinned to a final plant density of 65,250 plants ha^−1^ with 15 plants per row. A total of 160 plants were divided into two parts. The first part containing 100 plants was used to prepare the samples for sequencing; the second part containing 60 plants was used to measure the IL of each internode. Based on previous studies on maize internodes development [38], the six internodes of these two inbred lines were sampled to ensure they were in the equivalent developmental stages.

When the 7^th^, 8^th^ and 9^th^ internode of the two inbred lines reached 10 mm, the internode was removed from each plant and immediately frozen in liquid nitrogen for further studies. Six different samples for each internode were mixed for the RNA extractions. Total RNAs were isolated from these samples using TRIzol reagent (Invitrogen, Carlsbad, CA, USA) according to the manufacturer’s instructions. The total RNA’s quantity and purity were determined using a Bioanalyzer 2100 and RNA 6000 Nano Lab Chip Kit (Agilent, CA, USA) with a RIN number >7.0. After pollination, PH, EH, IL and IN were measured in 60 plants in the field. The data were analyzed using the DPS software. One-way analyses of variance were used to determine the average IL. A value of P ≤ 0.05 was considered statistically significant.

### Small RNA Library Preparation and Sequencing

The samples of six internodes were selected for library construction, and 20 μg of total RNAs from each internode was supplied for Illumina deep sequencing (BGI, Beijing, China). Small RNAs of 18–30 nt in length were purified using polyacrylamide gel electrophoresis, and 3′ and 5′ adaptors were added to the RNA termini. The RNAs were used for cDNA synthesis. Then, the cDNAs were amplified and subjected to sequencing.

The sequencing data of miRNA and degradome in this study was deposited in SRA-Archive (http://www.ncbi.nlm.nih.gov/sra) under the accession numbers SRP087051 (miRNA) and SRP087126 (degradome).

### Data Analysis

The low quality tags and the tags shorter than 18 nt were filtered out of the raw Solexa sequencing data and the appropriate small RNA tags were obtained. The functional small RNAs were acquired after removing the tRNA, rRNA and snoRNAs reads. Among the remaining small RNAs, conserved miRNA members, those previously reported in plant species, were selected to compare with the reported maize miRNAs in miRBase (http://www.miRBase.org). The comparable reading frequencies of a given miRNA in the different internodes of the two inbred lines were calculated by reads per million.

To discover the novel miRNAs, the un-annotated small RNAs were used as query and searched against the maize genome with the BLASTN algorithm (http://blast.ncbi.nlm.nih.gov/). Sequences that mapped to the maize genome were processed by the prediction software MIREAP (https://sourceforge.net/projects/mireap/). The strict criteria for the small RNAs to be regarded as miRNA candidate were as follows: the selected sequences could be folded into an appropriate stem-loop hairpin secondary structure, and a maximum of six unpaired nucleotides were allowed between the predicted mature miRNA sequence comprising one arm of the hairpin structure and its perfect complementary miRNA* sequence on the other arm of the hairpin. Additionally, the predicted secondary structure had a high minimal folding free energy index (MFEI) and a negative minimal folding free energy. Approximately 90% of miRNA precursors have an MFEI larger than 0.85, while the remaining have an MFEI lower than 0.85 [39].

### Target Gene Prediction

To predict the target genes of plant miRNAs, three internet-based computing systems, miRU (http://bioinfo3.noble.org/miRNA/miRU.htm), psRNATarget (http://plantgrn.noble.org/psRNATarget/) and helper tools (http://omicslab.genetics.ac.cn/psRobot/), were queried using the mature miRNA sequences. The targets that appeared in more than two sets were selected. Limited mismatches including no more than three nucleotides, indels no longer than one nucleotide and fewer than five G-U pairs were allowed between the miRNA and its potential target sequences. The predicted target genes were used queried using the BLASTN algorithm (http://blast.ncbi.nlm.nih.gov/) and against the GO database (http://www.geneontology.org/) for functional annotations.

### Degradome Sequencing

Two degradome cDNA libraries were constructed to identify the targets of the miRNAs. A mixture containing 20 μg RNA from three internodes of each inbred line was used to construct the degradome libraries. The method followed that of Addo-Quaye et al. [40] with some modifications. (1) Approximately 150 ng of poly (A)+ RNA was used as input RNA and annealed to biotinylated random primers. (2) Strapavid captured the RNA fragments using the biotinylation. (3) 5′ adaptors ligated to only those RNAs containing 5′ mono-phosphates. (4) Reverse transcription and PCR were performed. (5) Libraries were sequenced using the 5′ adapter only, resulting in the sequencing of the first 36 nt of the inserts that represented the 5′ ends of the original RNAs. Then, according to the vendor’s recommended protocol, single-end sequencing (36 bp) was performed on an Illumina Hiseq2500 at LC-BIO (Hangzhou, China).

Raw sequencing reads were obtained using Illumina’s Pipeline v1.5 software following a sequencing image analysis by the Pipeline Firecrest Module and base-calling by the Pipeline Bustard Module. The extracted sequencing reads were stored and then used in the standard data analysis. A public software package, CleaveLand3.0, was used for analyzing the generated sequencing data.

### Identification of miRNAs and Their Targets by qRT-PCR

To validate the candidate miRNAs, a polyA tail was ligated to the mature miRNAs 3′ end by polyA polymerase. The ligated products were used to start the reverse transcription with the Universal Adapter Primer and Prime ScriptRTase (containing oligo-dT) according to the supplier’s manual (Takara, Dalian China). The reverse transcription product was amplified using a miRNA-specific forward primer and a universal reverse primer.

A IQ5 Real Time PCR system (Bio-Rad, USA) was used to perform the qRT-PCR. The specific primers for qRT-PCR on the mature miRNAs were designed with the software Premier 5.0 (PREMIER Biosoft Int., Palo Alto, CA, USA). The PCR program was as follows: 95°C for 5 min; then 40 cycles (95°C for 15 s and 55°C for 30 s); and finally 40°C for10 min. The PCR primers for the selected mature miRNAs and their target genes are listed in S1 Table. Three biological replicates were performed for each internode. Technical repetitions were performed three times for each biological replicate, and 18S rRNA was the endogenous internal reference gene.

## Results

### The Different ILs from the 7^th^ to the 9^th^ Internode in the Two Inbred Lines

After pollination, the IL of each internode from the 7^th^ to the 9^th^ internode in the two inbred lines was measured. There were significant differences between any two internodes from the 7^th^ to the 9^th^ internodes in each inbred line and between the corresponding internodes in the two inbred lines (Table 1). The IL of each internode in the inbred line ‘Xun9058’ was much longer than it was in the corresponding internodes of ‘Xun928’. Additionally, the ILs in the two inbred lines increased in length gradually from the 7^th^ to the 9^th^ internode.

**Table 1:** Average IL (cm) of the 7^th^ to the 9^th^ internodes in ‘Xun928’ and ‘Xun9058’.

The internodes under ear	The Internode Length
The ninth internode of ‘Xun9058’	9.62±0.20 a
The ninth internode of ‘Xun928’	8.55±0.32 b
The eighth internode of ‘Xun9058’	7.85±0.15 b
The eighth internode of ‘Xun928’	6.79±0.30 c
The seventh internode of ‘Xun9058’	5.88±0.11 c
The seventh internode of ‘Xun928’	4.73±0.19 d

Data of each sample was the means from 60 plants. Different lower case letters in the column indicate significant differences at P ≤ 0.05 according to least significance difference tests. Data represent the mean values ± SE.

### Deep Sequencing of Maize Small RNAs

**Fig 1:**
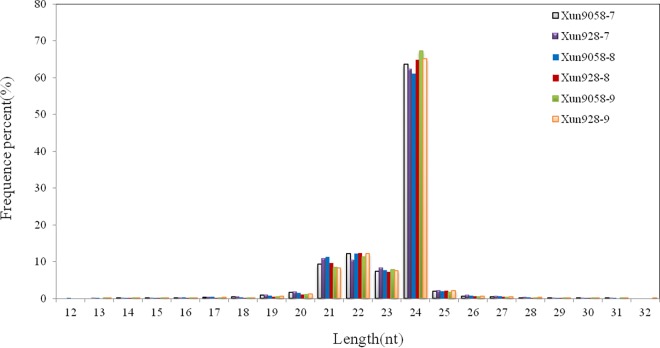
Length distribution of small RNAs obtained from the six maize internode libraries. The 24 nt was the most abundant size of the small RNAs, accounting for ~60% at each internode.

Deep sequencing of small RNAs was performed on the six internodes. Six miRNA libraries from the 7^th^, 8^th^ and 9^th^ internodes of the two inbred lines were independently analyzed using Solexa deep sequencing techniques (S2–S5 Tables). A total of 11,638,690, 15,012,692 and 16,055,896 reads were obtained from the 7^th^, 8^th^ and 9^th^ internodes, respectively, of the inbred line ‘Xun9058’. After removing the low quality, adaptor and short (< 18 nt) reads, 11,453,810, 14,720,104 and 15,834,866 reads, respectively, remained. The corresponding data in the inbred line ‘Xun928’ were 13,634,365, 13,887,119 and 13,574,902, respectively, with 13,415,671, 13,705,664 and 13,350,689 reads remaining, respectively. Then, all of the reads were aligned to the maize B73 genome. In total, 8,382,833, 10,923,963 and 11,500,050 corresponding to the 7^th^ to 9^th^ internodes, respectively, of the inbred line ‘Xun9058’ perfectly matched the maize genome, representing 73.19%, 74.21%, 72.62% of the total reads, respectively. And 9,162,866, 9,291,120 and 8,952,829 reads corresponding to the 7^th^ to 9^th^ internodes, respectively, of the inbred line ‘Xun928’ perfectly matched the maize genome, representing 68.30%, 67.79% and 67.06% of the total reads, respectively. Non-coding RNAs, including tRNAs, rRNAs, snRNAs and snoRNAs, accounted for 3.02%, 2.00% and 1.24%, of the total reads from the 7^th^ to 9^th^ internodes, respectively, in inbred line ‘Xun9058’, and 2.56%, 1.65% and 1.60% of the total reads from the 7^th^ to 9^th^ internodes, respectively, in inbred line ‘Xun928’, respectively. The sequences without non-coding reads were considered as small RNAs for the following analysis. The most abundant size of the small RNAs (Fig 1) was 24 nt, accounting for ~60% at each internode.

### Identification of Conserved Maize miRNAs and Candidate miRNAs

**Fig 2:**
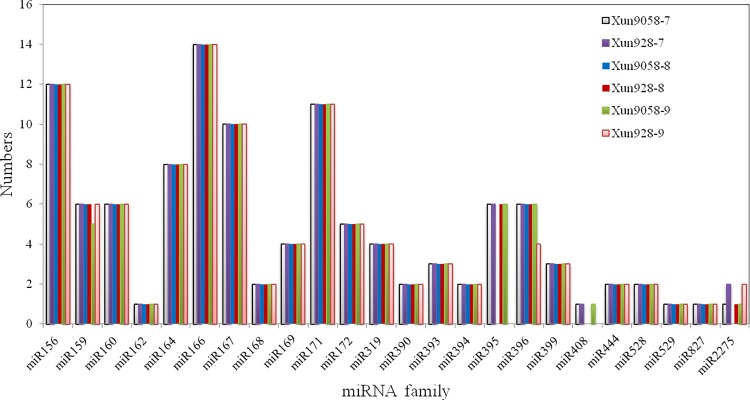
Conserved miRNA family members in the six maize internode libraries. miR166 consisted of 14 members, miR156 and miR167 had 12 and 10 members, respectively, and miR162, miR529, miR827 and miR1432 had only one member.

From the sequencing data, 609,602, 1,073,964 and 801,883 total reads from the 7^th^ to 9^th^ internodes, respectively, of the inbred line ‘Xun9058’ representing 481, 460 and 495 unique RNAs, respectively, which matched to maize miRNAs (S4 Table). The other data set from the inbred line ‘Xun928’ was similar to that of ‘Xun9058’ (S5 Table). Additionally, 21 conserved miRNA families comprised of 99 individual miRNAs, except those whose reads were less than two, were identified in all six libraries and similar abundance distributions were found in the conserved miRNA families (S1 Fig). miR168 was the most abundant family, followed by miR166. The least abundant miRNA families were miR160, miR394, miR529 and miR1432. In the identified miRNA families (Fig 2), miR166 consisted of 14 members, miR156 and miR167 had 12 and 10 members, respectively, and miR162, miR529, miR827 and miR1432 had only one member. However, miR482 and miR2118 were not detected in any of the six libraries.

To determine the differentially expressed miRNAs between different internodes, the comparable reading frequencies of miRNAs were calculated and normalized to the RPM (reads per million). The fold-changes and *p*-values of all the miRNAs were also calculated. miRNAs with *p*-values less than 0.05 and log2 fold changes>1 or <1 were designated as up-regulated or down-regulated. For the three internodes of ‘Xun9058’, compared with the expression of miRNAs in the 7^th^ internode, the numbers of up-regulated and down-regulated miRNA candidates were 2 and 26 in the 9^th^ internode, and 4 and 16 in the 8^th^ internode, respectively. Compared with the expression of miRNAs in the 8^th^ internode of ‘Xun9058’, the numbers of up-regulated and down-regulated miRNA candidates were 11 and 24 in the 9^th^ internode of ‘Xun9058’, respectively (S6 Table). Similarly, for the three internodes of ‘Xun928’, compared with the expression of miRNAs in the 7^th^ internode, the numbers of up-regulated and down-regulated miRNA candidates were 3 and 36 in the 9^th^ internode, and 13 and 13 in the 8^th^ internode, respectively. Compared with the expression of miRNA in the 8^th^ internode of ‘Xun928’, a total of 3 and 32 miRNAs were up-regulated and down-regulated in the 9^th^ internode of ‘Xun928’, respectively (S7 Table). Besides, a total of 31 miRNAs showed significant changes are common to the corresponding comparison groups of the 7^th^, 8^th^ and 9^th^ internodes of ‘Xun9058’ and ‘Xun928’(S8 Table). Compared with the expression of miRNAs in the 7^th^ and 8^th^ internode, miR156a-I and l were both down-regulated in the 9^th^ internode of ‘Xun9058’, the same as them in ‘Xun928’. miR160d, e and g were both down-regulated in the 8^th^ internode by comparing with it in the 7^th^ internode of two inbred lines, respectively. The expression changes of miR164e, f, h, miR319a-d, miR393a, c, miR396a-d, miR399e, I, j and miR528a, b also showed similarities between the corresponding comparison groups of the 7^th^, 8^th^ and 9^th^ internode of ‘Xun9058’ and ‘Xun928’.

For the corresponding internodes of ‘Xun9058’ and ‘Xun928’, compared with the expression of miRNAs in the 7^th^, 8^th^ and 9^th^ internodes of ‘Xun928’, the numbers of up-regulated and down-regulated miRNAs were 11 and 36 in the 7^th^ internode, 9 and 45 in the 8^th^ internode, and 9 and 25 in the 9^th^ internode of ‘Xun9058’, respectively (S9 Table). Moreover, 10 miRNA families containing 45 members showed significant changes at least in two internodes of ‘Xun928’ by comparing with the corresponding internodes of ‘Xun9058’ (S10 Table). Compared with the expression of miRNAs in the 7^th^, 8^th^ and 9^th^ internodes of ‘Xun928’, miR156a-I, l, miR167a-d, miR169r and miR396c, d were all down-regulated in the corresponding internodes of ‘Xun9058’; however, miR396a, b were up-regulated in the corresponding internodes of ‘Xun9058’. miR160a-e, g and miR164e were both up-regulated in the 7^th^ internode of ‘Xun9058’, whereas they were down-regulated in the 8^th^ internode of ‘Xun9058’ by comparing with them in the corresponding internodes of ‘Xun928’. The expression of miR164a-g, miR167e, f, j, miR172a-d, miR393a, miR399e, I, j and miR528a, b also showed significant changes in two corresponding internodes of ‘Xun9058’ and ‘Xun928’.

### Prediction of Novel Maize miRNAs

Among the novel miRNAs predicted, 4 members from 3 miRNA families were co-detected in the six internodes of two inbred lines (S11 Table). All of these miRNA precursors had a high minimal folding free energy index (MFEI) larger than 0.85. Among these sequences, 21nt were the most abundant fractions, which was different from the length distribution of conserved small RNAs detected. The length of the novel miRNAs precursors varied from 104 to 178 nt. The free energy varied from -53.4 kcal mol^-1^ to -98.5 kcal mol^-1^. For the three internodes of ‘Xun9058’, the expression of the novel miRNAs showed no significant changes between any two internodes (S12 Table). The expression of the novel miRNAs also showed no significant changes between any two internodes of ‘Xun928’ (S13 Table). For the corresponding internode of ‘Xun9058’ and ‘Xun928’, only the expression of miRNAn1 was up-regulated in the 7^th^ and 8^th^ internodes of ‘Xun9058’ compared with it in the corresponding internodes of ‘Xun928’ (S14 Table).

### Identification of miRNA Targets by Degradome Sequencing

To understand the function and potential regulatory roles of the identified miRNAs involved in the 7^th^ to 9^th^ internodes of each inbred line, two degradome cDNA libraries (IUE_X928 and IUE_X9058) were constructed to identify the miRNA targets. In plants, miRNA mediated mRNA cleavage is highly specific, and miRNAs have been shown to bind with near perfect complementarity to their mRNA targets, which generally leads to the slicing of the mRNA between positions 10 and 11 of the Argonaute bound miRNA [41]. In this study, CleaveLand, a general pipeline, was applied to detect fragments diagnostic of small RNA-mediated cleavage from degradome sequencing experiments for conserved miRNAs in the internodes of two inbred lines [40]. In total, 102 and 120 target transcripts representing 16 and 20 different miRNAs families were detected by degradome sequencing in the two inbred lines ‘Xun928’ and ‘Xun9058’, respectively. These target genes were further annotated by GO analysis. Based on the analysis of the conserved miRNAs (S8 and S10 Tables), a total of 58 target transcripts of the candidate miRNAs were all picked out to illuminate the roles of the identified miRNAs (S15 Table). However, the target transcripts of miR164e and miR528a,b were not detected in any degradome libraries. The remaining targets were detected in both degradome libraries and represented 11 different miRNAs families. The cleavage site of mRNA targets occurs exactly between the 10th and 11th nucleotide of complementarity relative to the small RNA. Most of the targets were found to be transcription factors (TFs), such as *auxin response factors* (miR160 and miR167), *growth-regulating factor* (miR396), *N-acetylcysteine domain containing protein* (miR164), *SQUAMOSA promoter-binding protein-like* (miR156) and *nuclear TF Y* (miR169). Additionally, it was common to find multiple genes targeted by single miRNAs and a single gene regulated by multiple miRNAs. For instance, each member of miR156 targeted eight genes and all 12 members of miR156 regulated the target GRMZM2G097275_T01. The targets of the miR160 and miR393 families belonged to the TF genes of the *ARFs* and *auxin signaling F-BOX 2* (*AFB2*) families, respectively. However, other miRNAs cleaved targets belonging to different gene families. For instance, the miR156 family had different targets, including *SQUAMOSA promoter-binding protein-like* and *histidine-containing phosphotransfer factor 5*. The genes encoding *ARFs* were identified as the targets of miR160 and miR167. Most of the target genes were involved in a wide spectrum of regulatory functions and biological processes, including the regulation of transcription, DNA binding and responses to hormone stimuli. *NACs* and *NF-Y* were the main targets of the miR164 and mi169 families.

### Expression Profiles of miRNAs and Target Genes

**Fig 3:**
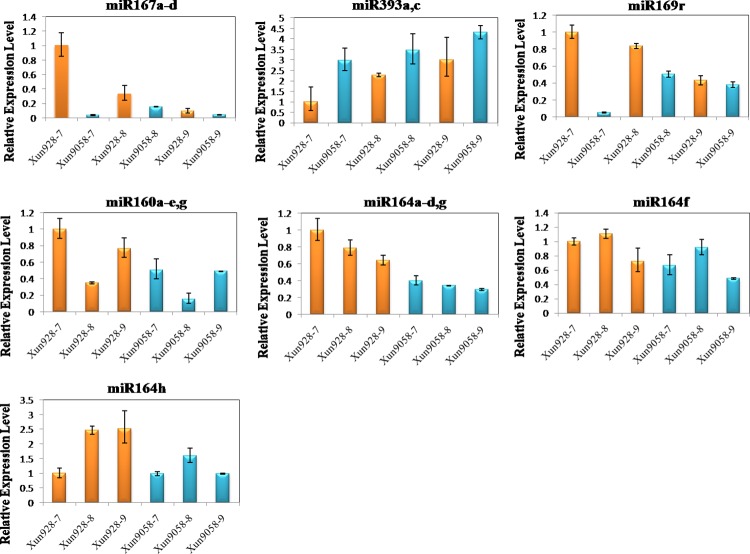
Expression levels of maize internode-associated miRNAs as determined by RT-PCR. 18S rRNA was used as the reference gene. Three biological replicates and three technical replicates were performed. Data represent the mean values ± SD of three replicates.

**Fig 4:**
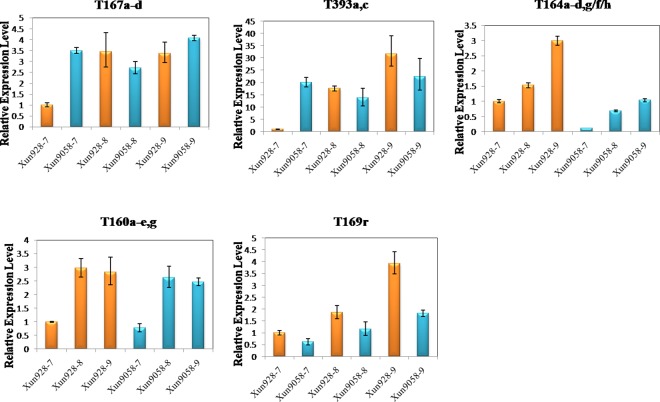
Expression levels of the maize internode-associated miRNA targets as determined by RT-PCR. 18S rRNA was used as the reference gene. Three biological replicates and three technical replicates were performed. T, target. Data represent the mean values ± SD of three replicates.

Based on the analysis of the sequencing data, five miRNA families (five known miRNAs families) and their targets were selected to detect their expression levels in developing internodes using qRT-PCR (Figs 3 and 4). The expression changes of the miRNAs established using qRT-PCR were consistent with the data analysis results on the three internodes of each inbred line or in the corresponding internodes of the two inbred lines, except zma-miR164h and zma-miR393a, c in the three internodes of each inbred line, and zma-miR160a-e, g in the corresponding internodes of the two inbred lines. The expression level of the target of zma-miR160 was negatively correlated with the miRNA levels in the7^th^ to 9^th^ internodes of the two inbred lines. The expression level of zma-miR169r was negatively correlated with its targets between the corresponding internodes of two inbred lines. However, the expression level of the targets of zma-miR164f, h, zma-miR167, zma-miR169 and zma-miR393 were not negatively correlated with the miRNA levels in the 7^th^ to 9^th^ internodes of the two inbred lines. According to the degradome sequencing data, only one target was detected for the different members of zma-miR160. However, the miRNAs zma-miR164, zma-miR167, zma-miR169 and zma-miR393 had multiple members and targeted multiple genes. This might explain the negative or non-negative correlative relationships between the miRNAs and their targets. The results indicated a complex regulatory relationship between miRNAs and their targets.

## Discussion

### Importance of Research on the IL under the Maize Ears

In maize, the EH is a very important characteristic for breeding. Hybrids or inbred lines with high EHs often undergo lodging in the field, resulting in a loss of grain yield [4]. In a previous study, PH showed a high correlation with EH [6], and they were mainly composed of ILs and INs. For different hybrids and inbred lines, the internodes gradually elongated from the 6^th^ internode to the ear. The 7^th^ to 9^th^ internodes always elongated rapidly, and their lengths were significant correlated with EH and PH. In a previous study, Tang et al. [37] reported that the IL was the main contributor to PH and EH in a maize recombinant inbred line population, and that the *br2* gene had been proven to shorten the ILs of lower stalk [13]. In this study, we found that the ILs of the inbred lines ‘Xun928’ and ‘Xun9058’ also increased gradually from the 7^th^ to the 9^th^ internode (Table 1), and the two inbred lines had significant IL differences from the 7^th^ to the 9^th^ internode. The study about the ILs under the ear will help to select the lodging-resistance Hybrids and inbred lines. However, only few researches have been reported about this phenotype. The regulation mechanism of the ILs under the ear is still not completely clear. It is the first time to identify miRNAs and their targets involved in the developing internodes under the ear in the present study.

### miRNAs and Their Targets Involved in the Development of Internodes under Maize Ear

In monocots, the internode elongation is attributed to the development of intercalary meristems at the base of the growing internode, which are capable of cell division and cell elongation [42,43]. Numerous studies have shown that plant organ growth that is attributable to cell division and turgor-driven wall expansion, is to a large extent regulated by internal signaling molecules (phytohormones) and environmental cues (light, gravity, etc.), which must be integrated at intracellular and organismic levels[44–46]. In Arabidopsis and rice, the regulatory factors, brassinosteroids and CKs, play important roles in PH regulation. However, in maize, GA_3_ and IAA are the primary determining factors of PH [8,47–52]. *D8* is a negative GA-responsive regulator in maize [8]. *BR2* functions in IAA export from intercalary meristems via a monocot-specific mechanism [53]. In addition to the key genes regulating the hormone responses that determine EH and PH, TFs are also the main regulators in hormone-signaling responses. In this study, five miRNA families and their targets belonging to TFs were selected to illustrate the roles of miRNAs involved in the developing internodes under the ear.

*ARFs* are the important TFs in the auxin signaling pathway, regulating the transcription of auxin-responsive genes by directly binding to the auxin response element (TGTCTC) in their promoters [54]. The release of ARF repression forms a paradigm of the transcriptional response to auxin in the presence of auxin by degrading their cognate Aux/IAA repressors [55]. Different ARF proteins regulate diverse developmental stages, such as embryogenesis, root development and floral organ formation [18,56–58]. Mutations in MP/ARF5, ARF6, NPH4/ARF7, ARF8 and ARF19 decrease the induction of auxin-responsive genes and cause auxin-related developmental defects at various stages of development. The *mp/arf5* mutants have defects in embryonic and vascular patterning [59,60]. In Arabidopsis, *arf6* and *arf8* single mutant plants have delayed stamen development and decreased fecundity, whereas *arf6 arf8* double mutant plants have a complete block in flower maturation [61]. Mutations in the *ARF19* gene can decrease auxin-induced gene activation by combining with mutations in *ARF7*/*NPH4*, and the *arf7/nph4arf19* double mutants make very few lateral or adventitious roots and have small leaves [62]. The *arf8-1* mutant gene showed an elongated-hypocotyl phenotype under light conditions [63]. Additionally, *OsARF12*, a transcription activator of the auxin response gene, regulates root elongation and affects iron accumulation in rice (*Oryza sativa* L.) [19]. In this study, *ARF6* and *ARF8* were predicted to be the targets of miR167a-d, whereas ARF16 was the target of miR160a-e, g. In the 7^th^ and the 9^th^ internodes of ‘Xun9058’, the expression level of the miR167 target was much higher than it in the corresponding internodes of ‘Xun928’, and miR167 negatively regulated its target. Furthermore, the expression level of the miR160 target showed a similar variation trend from the 7^th^ to the 9^th^ internode between ‘Xun928’ and ‘Xun9058’. The ARFs might promote the elongation and development of the corresponding internodes under the maize ear between two inbred lines or different internodes under the maize ear from the same inbred line by activating the auxin-response genes, and they were regulated by miRNA167 and miRNA160, respectively.

*Aux/IAA* genes function as transcriptional repressors of early auxin-responsive gene expression and the Aux/IAA proteins contain four conserved domains (I, II, III and IV). Domain II plays a role in destabilizing Aux/IAA proteins and is the site of interaction with the F-box protein TRANSPORT INHIBITOR RESPONSE/AUXIN SIGNALING F-BOX PROTEIN (TIR1/AFB) [64,65]. The TIR1 F-box protein, acting as an auxin receptor, contains five additional AFB proteins, AFB1 to AFB5, and directly links auxin perception to the degradation of the Aux/IAA proteins [66,67]. *AFB2* is a TIR1 protein and an important TF. The relationship between auxin and *AFB2* has also been reported to be modulated by IAA gene expression levels through direct physical interactions with the AFBs’ auxin-receptor proteins, resulting in the targeted degradation of Aux/IAA transcriptional repressor proteins via the Skp, Cullin, F-box protein complex E3-ubiquitin ligase proteasome pathway [68]. TIR1 and AFB2 groups act as positive regulators of auxin signaling [69,70]. The miR393 has been implicated in down-regulating the expression of *TIR1*/*AFB* genes in Arabidopsis [22,23]. In this study, AFB2 was predicted to be the target of miR393. The expression levels of the miR393 target were higher in the 8^th^ and the 9^th^ internodes of ‘Xun928’ than in the corresponding internodes of ‘Xun9058’. The miR393 target was negatively regulated by miR393 in these two internodes. miR393 might reduce the expression of its target, thereby promoting the elongation and development in the corresponding internodes under the ears of different inbred lines in maize.

*NACs* are a large group of plant-specific TFs that play important roles in a diverse set of developmental processes. *NAC1* belongs to the *NAC* family [71,72]. In Arabidopsis, *NAC1* is induced by auxin and mediates auxin signaling to promote lateral root development [71]. Additionally, auxin induces *NAC1* expression at a reduced level in leaves and stems [72–74]. miR164 in the late auxin response was intended to clear *NAC1* mRNA, which would attenuate the auxin signaling that inhibits lateral root development [20]. In this study, *NAC1* was predicted by degradome sequencing to be the target of miR164. Its expression level also showed a gradual increase from the 7^th^ to the 9^th^ internode in both inbred lines, and *NAC1* was negatively regulated by miR164a-d, and g. miR164 might decrease the expression of *NAC1* by responding to auxin signaling, thereby reducing the elongation and development of the different internodes of the same inbred lines.

NF-Y TFs, which are composed of three subunits (NF-YA, NF-YB and NF-YC), are capable of the highly specific transcriptional regulation of target genes by binding to the CCAAT-containing promoter sequences [75]. Several recent publications have demonstrated that NF-YA subunits regulate the expression of the core ABA signaling components and play essential roles during ABA-mediated responses in plants [76,77]. The over expression of *GmNFYA3*, a target gene of miR169, resulted in reduced leaf water loss and enhanced drought tolerance in Arabidopsis. The transcript levels of ABA biosynthesis (ABA1 and ABA2) and ABA signaling (ABI1 and ABI2) were generally higher in *GmNFYA3* plants than in wild-type controls under normal conditions [21]. NF-YA3, NF-YA5 and NF-YA6 were all targets of miR169 in this study. The expression levels of the miR169 targets were up-regulated in the 7^th^, 8^th^ and 9^th^ internodes in ‘Xun9058’ compared with in the corresponding internodes of ‘Xun928’. miR169r negatively affected the expression levels of the target in these internodes. It might inhibit the elongation and development in corresponding internodes under the ear of different inbred lines.

In conclusion, 20 miRNAs belonging to five miRNA families were select to illustrate their roles involved in the internode elongation and development. These miRNAs might regulate the internode elongation and development by their targets respond to hormone signaling, thereby formed the final ILs. The involvement of miRNAs and their targets in the internodes elongation and development provided the possible regulation mechanism of the ILs under the ear. Although the mechanism how the miRNAs promote or inhibit the internode elongation is not well known, our results provided a valuable reference for understanding the possible regulation mechanism of the ILs under the ear. Further research is also needed to identify the regulation mechanism how the internodes reached their final significantly different ILs.

## Supporting Information

S1 Fig Conserved miRNA abundance levels in the six libraries. (TIF)

S1 Table Primers used to amplify mature miRNAs and their targets using qRT-PCR. (DOCX)

S2 Table Statistical analysis of sequencing reads in the three internode libraries of maize ‘Xun9058’. (DOCX)

S3 Table Statistical analysis of sequencing reads in the three internode libraries of maize ‘Xun928’. (DOCX)

S4 Table Distribution of the small RNA sequences in the three internode libraries of maize ‘Xun9058’. (DOCX)

S5 Table Distribution of the small RNA sequences in the three internode libraries of maize ‘Xun928’. (DOCX)

S6 Table The conserved miRNAs showed significant changes for each pairwise comparison among the 7^th^, 8^th^ and 9^th^ internodes of ‘Xun9058’. (DOCX)

S7 Table The conserved miRNAs showed significant changes for each pairwise comparison among the 7^th^, 8^th^ and 9^th^ internodes of ‘Xun928’. (DOCX)

S8 Table The conserved miRNAs showed significant changes are common to the corresponding comparison groups of the 7^th^, 8^th^, and 9^th^ internodes of ‘Xun9058’ and ‘Xun928’. (DOCX)

S9 Table The conserved miRNAs showed significant changes between the corresponding internodes of ‘Xun9058’ and ‘Xun928’. (DOCX)

S10 Table The conserved miRNAs showed significant changes at least in two internodes of ‘Xun928’ compared with the corresponding internodes of ‘Xun9058’. (DOCX)

S11 Table Novel miRNAs co-detected in six internodes libraries. (DOCX)

S12 Table The expression changes of novel miRNAs for each pairwise comparison among the 7^th^, 8^th^ and 9^th^ internodes of ‘Xun9058’. (DOCX)

S13 Table The expression changes of novel miRNAs for each pairwise comparison among the 7^th^, 8^th^ and 9^th^ internodes of ‘Xun928’. (DOCX)

S14 Table The expression changes of novel miRNAs between the corresponding internodes of ‘Xun9058’ and ‘Xun928’. (DOCX)

S15 Table Internode development associated with maize internode miRNA target genes detected via a genome wide degradome. (DOCX)
