# Supplementary material for: Genome-Wide Identification of miRNAs and Their Targets Involved in the Developing Internodes under Maize Ears by Responding to Hormone Signaling
Source: PLoS One. 2016 Oct 3;11(10):e0164026. doi: 10.1371/journal.pone.0164026 (PMC5047619; doi:10.1371/journal.pone.0164026)
Supplement: S1 Table — (DOCX) [file pone.0164026.s002.docx]

**S1 Table.** **Primers used to amplify mature miRNAs and their targets using qRT-PCR.**

| Name | Primer | Sequence (5'-3') |
| --- | --- | --- |
| zma-miR160a-e,g | Forward primer | tGCCtGGCtCCCtGtAtGCC |
| zma-miR164a-d,g | Forward primer | tGGAGAAGCAGGGCACGtGCA |
| zma-miR164f | Forward primer | tGGAGAAGCAGGGCACGtGC |
| zma-miR164h | Forward primer | tGGAGAAGCAGGGCACGtGtG |
| zma-miR167a-d | Forward primer | tGAAGCtGCCAGCAtGAtCt |
| zma-miR169r | Forward primer | CAGCCAAGGAtGACttGCCGG |
| zma-miR393a,c | Forward primer | tCCAAAGGGAtCGCAttGAtCt |
| T160a-e,g(GRMZM2G159399_T01)-S |  | CGGCTTGGTGCTATGA |
| T160a-e,g(GRMZM2G159399_T01)-A |  | GCTTTCCGAACAGCATTA |
| T164a-d,g/f/h(GRMZM2G063522_T01 )-S |  | AGCGATGCGGATCGAAAT |
| T164a-d,g/f/h(GRMZM2G063522_T01 )-A |  | GGCATAGGGCATACAGACAT |
| T167a-d (GRMZM2G078274_T03)-S |  | ACCGCAGGAGAAAGGC |
| T167a-d (GRMZM2G078274_T03)-A |  | CTCAGCCACATCAAAGCA |
| T169r(GRMZM2G000686_T01)-S |  | CAAGAAGCGTCTGGAAT |
| T169r(GRMZM2G000686_T01)-A |  | AGGTTGCCTGAATGGTC |
| T393a,c(GRMZM2G137451_T01)-S |  | TGGCACGGTGGATTGT |
| T393a,c(GRMZM2G137451_T01)-A |  | TCCCGACAGTTCTCAGGTT |
| 18S-A |  | CCTGCGGCTTAATTGACTC |
| 18S-S |  | GTTAGCAGGCTGAGGTCTCG |
