# Supplementary material for: Genome-Wide Identification of miRNAs and Their Targets Involved in the Developing Internodes under Maize Ears by Responding to Hormone Signaling
Source: PLoS One. 2016 Oct 3;11(10):e0164026. doi: 10.1371/journal.pone.0164026 (PMC5047619; doi:10.1371/journal.pone.0164026)
Supplement: S2 Table — (DOCX) [file pone.0164026.s003.docx]

**S2 Table. Statistical analysis of sequencing reads in the three internode libraries of maize ‘Xun9058’.**

| Type | 9058-7count | Percent of total reads(%) | 9058-8count | Percent of total reads(%) | 9058-9count | Percent of total reads(%) |
| --- | --- | --- | --- | --- | --- | --- |
| total_reads | 11638690 |  | 15012692 |  | 16055896 |  |
| high_quality | 11563224 | 100% | 14905970 | 100% | 15940801 | 100% |
| 3'adapter_null | 23280 | 0.20% | 28485 | 0.19% | 36677 | 0.23% |
| insert_null | 1044 | 0.01% | 2509 | 0.02% | 3057 | 0.02% |
| 5'adapter_contaminants | 5562 | 0.05% | 10232 | 0.07% | 7873 | 0.05% |
| smaller_than_18nt | 79227 | 0.69% | 144349 | 0.97% | 57873 | 0.36% |
| polyA | 301 | 0.003% | 291 | 0.002% | 455 | 0.003% |
| clean_reads | 11453810 | 99.05% | 14720104 | 98.75% | 15834866 | 99.34% |
