# Supplementary material for: Genome-Wide Identification of miRNAs and Their Targets Involved in the Developing Internodes under Maize Ears by Responding to Hormone Signaling
Source: PLoS One. 2016 Oct 3;11(10):e0164026. doi: 10.1371/journal.pone.0164026 (PMC5047619; doi:10.1371/journal.pone.0164026)
Supplement: S3 Table — (DOCX) [file pone.0164026.s004.docx]

**S3 Table.** **Statistical analysis of sequencing reads in the three internode libraries of maize ‘Xun928’.**

| Type | 928-7count | Percent of total reads(%) | 928-8count | Percent of total reads(%) | 928-9count | Percent of total reads(%) |
| --- | --- | --- | --- | --- | --- | --- |
| total_reads | 13634365 |  | 13887119 |  | 13574902 |  |
| high_quality | 13546024 | 100% | 13795143 | 100% | 13482965 | 100% |
| 3'adapter_null | 23741 | 0.18% | 29858 | 0.22% | 30184 | 0.22% |
| insert_null | 1079 | 0.01% | 1240 | 0.01% | 1530 | 0.01% |
| 5'adapter_contaminants | 6611 | 0.05% | 7153 | 0.05% | 6017 | 0.04% |
| smaller_than_18nt | 98561 | 0.73% | 50824 | 0.37% | 94167 | 0.70% |
| polyA | 361 | 0.003% | 404 | 0.003% | 378 | 0.003% |
| clean_reads | 13415671 | 99.04% | 13705664 | 99.35% | 13350689 | 99.02% |
