# Supplementary material for: Genome-Wide Identification of miRNAs and Their Targets Involved in the Developing Internodes under Maize Ears by Responding to Hormone Signaling
Source: PLoS One. 2016 Oct 3;11(10):e0164026. doi: 10.1371/journal.pone.0164026 (PMC5047619; doi:10.1371/journal.pone.0164026)
Supplement: S4 Table — (DOCX) [file pone.0164026.s005.docx]

**S4 Table. Distribution of the small RNA sequences in the three internode libraries of maize ‘Xun9058’.**

| small RNA matching protein-coding genes | 9058-7U | 9058-7T | 9058-8U | 9058-8T | 9058-9U | 9058-9T |
| --- | --- | --- | --- | --- | --- | --- |
| exon_antisense | 43016（0.89%） | 102904(0.90%) | 40580(0.71%) | 86756(0.59%) | 59927(0.92%) | 145462(0.92%) |
| exon_sense | 73142（1.51%） | 154043(1.34%) | 66638(1.17%) | 150446(1.02%) | 89062(1.37%) | 202533(1.28%) |
| intron_antisense | 92970(1.92%) | 279964(2.44%) | 97125(1.70%) | 239830(1.63%) | 124062(1.91%) | 337943(2.13%) |
| intron_sense | 144110(2.98%) | 397463(3.47%) | 150481(2.63%) | 476987(3.24%) | 187945(2.90%) | 560204(3.54%) |
| non-coding RNAs |  |  |  |  |  |  |
| snoRNA | 790(0.02%) | 2295(0.02%) | 1021(0.02%) | 3042(0.02%) | 1063(0.02%) | 3257(0.02%) |
| snRNA | 2033(1.50%) | 4538(0.04%) | 2490(0.04%) | 6325(0.04%) | 2371(0.04%) | 5740(0.04%) |
| tRNA | 7877(0.16%) | 201996(1.76%) | 8790(0.15%) | 264434(1.80%) | 8367(0.13%) | 166225(1.05%) |
| rRNA | 64768(1.34%) | 556149(4.86%) | 101919(1.78%) | 894131(6.07%) | 68570(1.06%) | 506240(3.20%) |
| repeat | 1440912(29.79%) | 3603609(31.46%) | 1384084(24.21%) | 3872645(26.31%) | 1928634(29.72%) | 5149111(32.52%) |
| miRNAs |  |  |  |  |  |  |
| known | 481(0.01%) | 609602(5.32%) | 460(0.01%) | 1073964(7.30%) | 495(0.01%) | 801883(5.06%) |
| novel | 165(0.003%) | 14219(0.12%) | 211(0.004%) | 14783(0.10%) | 164(0.003%) | 20360(0.13%) |
| other small RNAs | 2966395(61.33%) | 5527028(48.25%) | 3862692(67.57%) | 7636761(51.88%) | 4019619(61.93%) | 7935908(50.12%) |
|  | 4836659 | 11453810 | 5716491 | 14720104 | 6490279 | 15834866 |
