# Supplementary material for: Genome-Wide Identification of miRNAs and Their Targets Involved in the Developing Internodes under Maize Ears by Responding to Hormone Signaling
Source: PLoS One. 2016 Oct 3;11(10):e0164026. doi: 10.1371/journal.pone.0164026 (PMC5047619; doi:10.1371/journal.pone.0164026)
Supplement: S5 Table — (DOCX) [file pone.0164026.s006.docx]

**S5 Table. Distribution of the small RNA sequences in the three internode libraries of maize ‘Xun928’.**

| small RNA matching protein-coding genes | 928-7U | 928-7T | 928-8U | 928-8T | 928-9U | 928-9T |
| --- | --- | --- | --- | --- | --- | --- |
| exon_antisense | 38281(0.73%) | 85973(0.64%) | 38019(0.73%) | 94394(0.69%) | 39558(0.73%) | 94280(0.71%) |
| exon_sense | 62773(1.20%) | 129924(0.97%) | 63710(1.22%) | 144903(1.06%) | 65716(1.21%) | 139210(1.04%) |
| intron_antisense | 86565(1.65%) | 281036(2.09%) | 85884(1.65%) | 276678(2.02%) | 89596(1.65%) | 283562(2.12%) |
| intron_sense | 135588(2.58%) | 391256(2.92%) | 133117(2.55%) | 421012(3.07%) | 139458(2.57%) | 410287(3.07%) |
| non-coding RNAs |  |  |  |  |  |  |
| snoRNA | 1228(0.02%) | 2925(0.02%) | 757(0.01%) | 2123(0.02%) | 719(0.01%) | 1944(0.01%) |
| snRNA | 2665(0.05%) | 6988(0.05%) | 2237(0.04%) | 5990(0.04%) | 2171(0.04%) | 4982(0.04%) |
| tRNA | 10079(0.19%) | 254234(1.90%) | 8800(0.17%) | 160949(1.17%) | 10811(0.20%) | 149463(1.12%) |
| rRNA | 120308(2.29%) | 984902(7.34%) | 74199(1.42%) | 689263(5.03%) | 73330(1.35%) | 549076(4.11%) |
| repeat | 1344449(25.62%) | 3566376(26.58%) | 1342044(25.72%) | 3920189(28.60%) | 1405010(25.90%) | 3959550(29.62%) |
| miRNAs |  |  |  |  |  |  |
| known | 508(0.01%) | 917560(6.84%) | 498(0.01%) | 797548(5.82%) | 445(0.01%) | 589385(4.41%) |
| novel | 186(0.004%) | 11421(0.09%) | 197(0.004%) | 16417(0.12%) | 171(0.003%) | 16760(0.13%) |
| other small RNAs | 3445688(65.65%) | 6783076(50.56%) | 3467439(66.47%) | 7176198(52.36%) | 3597907(66.32%) | 7152190(53.57%) |
|  | 5248318 | 13415671 | 5216901 | 13705664 | 5424892 | 13350689 |
