# Supplementary material for: Genome-Wide Identification of miRNAs and Their Targets Involved in the Developing Internodes under Maize Ears by Responding to Hormone Signaling
Source: PLoS One. 2016 Oct 3;11(10):e0164026. doi: 10.1371/journal.pone.0164026 (PMC5047619; doi:10.1371/journal.pone.0164026)
Supplement: S6 Table — (DOCX) [file pone.0164026.s007.docx]

**S6 Table. The conserved miRNAs showed significant changes for each pairwise comparison among the 7^th^, 8^th^ and 9^th^ internodes of ‘Xun9058’.**

|  | RPM | RPM | RPM | Log2 | Log2 | Log2 |
| --- | --- | --- | --- | --- | --- | --- |
| miRNA | 9058-7 | 9058-8 | 9058-9 | 9058-9/9058-7 | 9058-9/9058-8 | 9058-8/9058-7 |
| zma-miR156a | 63.6469 | 71.5348 | 21.8505 | -1.54 | -1.71 | - |
| zma-miR156b | 124.9366 | 137.1594 | 41.7433 | -1.58 | -1.72 | - |
| zma-miR156c | 63.6469 | 71.5348 | 21.8505 | -1.54 | -1.71 | - |
| zma-miR156d | 127.5558 | 138.5180 | 44.9641 | -1.50 | -1.62 | - |
| zma-miR156e | 63.1231 | 71.1951 | 21.9768 | -1.52 | -1.70 | - |
| zma-miR156f | 62.8612 | 70.4479 | 21.7242 | -1.53 | -1.70 | - |
| zma-miR156g | 62.8612 | 70.4479 | 21.7242 | -1.53 | -1.70 | - |
| zma-miR156h | 63.1231 | 71.1951 | 21.9768 | -1.52 | -1.70 | - |
| zma-miR156i | 63.1231 | 71.1951 | 21.9768 | -1.52 | -1.70 | - |
| zma-miR156j | 19.2076 | 7.8804 | 17.9983 | - | 1.19 | -1.29 |
| zma-miR156l | 63.1231 | 71.1951 | 21.9768 | -1.52 | -1.70 | - |
| zma-miR159a | 354.8164 | 536.6810 | 194.4443 | - | -1.46 | - |
| zma-miR159b | 354.3799 | 535.1185 | 194.0023 | - | -1.46 | - |
| zma-miR159f | 354.8164 | 536.6810 | 194.3180 | - | -1.47 | - |
| zma-miR159j | 354.3799 | 535.0506 | 193.8760 | - | -1.46 | - |
| zma-miR159k | 354.3799 | 535.0506 | 193.8760 | - | -1.46 | - |
| zma-miR160a | 3.0558 | 0.1359 | 0.2526 | -3.60 | - | -4.49 |
| zma-miR160b | 3.0558 | 0.1359 | 0.2526 | -3.60 | - | -4.49 |
| zma-miR160c | 3.0558 | 0.1359 | 0.2526 | -3.60 | - | -4.49 |
| zma-miR160d | 3.0558 | 0.1359 | 0.3158 | -3.27 | 1.22 | -4.49 |
| zma-miR160e | 3.0558 | 0.1359 | 0.3158 | -3.27 | 1.22 | -4.49 |
| zma-miR160g | 3.0558 | 0.1359 | 0.3158 | -3.27 | 1.22 | -4.49 |
| zma-miR164e | 4.4527 | 0.9511 | 1.8314 | -1.28 | - | -2.23 |
| zma-miR164f | 13.4453 | 9.9184 | 6.2520 | -1.10 | - | - |
| zma-miR164h | 1.3096 | 0.6793 | 0.4421 | -1.57 | - | - |
| zma-miR171h | 1.1350 | 0.5435 | 1.3893 | - | 1.35 | -1.06 |
| zma-miR171k | 1.1350 | 0.5435 | 1.3893 | - | 1.35 | -1.06 |
| zma-miR171l | 0.1746 | 0.5435 | 0.4421 | 1.34 | - | 1.64 |
| zma-miR171m | 0.1746 | 0.5435 | 0.4421 | 1.34 | - | 1.64 |
| zma-miR319a | 29.6844 | 1.4946 | 9.9780 | -1.57 | 2.74 | -4.31 |
| zma-miR319b | 33.5260 | 3.2608 | 12.5672 | -1.42 | 1.95 | -3.36 |
| zma-miR319c | 29.5971 | 1.3587 | 9.9780 | -1.57 | 2.88 | -4.45 |
| zma-miR319d | 33.5260 | 3.2608 | 12.5672 | -1.42 | 1.95 | -3.36 |
| zma-miR393a | 7.7703 | 2.4456 | 4.6101 | - | - | -1.67 |
| zma-miR393c | 3.2304 | 1.4266 | 1.7051 | - | - | -1.18 |
| zma-miR396a | 1.4842 | 5.7744 | 1.4525 | - | -1.99 | 1.96 |
| zma-miR396b | 1.4842 | 5.7744 | 1.4525 | - | -1.99 | 1.96 |
| zma-miR396c | 20.9537 | 27.1058 | 11.1779 | - | -1.28 | - |
| zma-miR396d | 20.9537 | 27.1058 | 11.1779 | - | -1.28 | - |
| zma-miR399e | 0.3492 | 0.3397 | 0.1263 | -1.47 | -1.43 | - |
| zma-miR399i | 0.3492 | 0.3397 | 0.1263 | -1.47 | -1.43 | - |
| zma-miR399j | 0.3492 | 0.3397 | 0.1263 | -1.47 | -1.43 | - |
| zma-miR528a | 22.4379 | 15.8287 | 33.3441 | - | 1.07 | - |
| zma-miR528b | 25.0572 | 16.8477 | 36.0597 | - | 1.10 | - |
| zma-miR529 | 3.8415 | 4.2119 | 2.0840 | - | -1.01 | - |

-: no significant changes.
