# Supplementary material for: Genome-Wide Identification of miRNAs and Their Targets Involved in the Developing Internodes under Maize Ears by Responding to Hormone Signaling
Source: PLoS One. 2016 Oct 3;11(10):e0164026. doi: 10.1371/journal.pone.0164026 (PMC5047619; doi:10.1371/journal.pone.0164026)
Supplement: S7 Table — (DOCX) [file pone.0164026.s008.docx]

**S7 Table. The conserved miRNAs showed significant changes for each pairwise comparison among the 7^th^, 8^th^ and 9^th^ internodes of ‘Xun928’.**

|  | RPM | RPM | RPM | Log2 | Log2 | Log2 |
| --- | --- | --- | --- | --- | --- | --- |
| miRNA | 928-7 | 928-8 | 928-9 | 928-9/928-7 | 928-9/928-8 | 928-8/928-7 |
| zma-miR1432 | 0.9690 | 1.3863 | 4.9436 | 2.35 | 1.83 | - |
| zma-miR156a | 228.5387 | 215.0936 | 97.2235 | -1.23 | -1.15 | - |
| zma-miR156b | 725.1967 | 581.5114 | 184.5598 | -1.97 | -1.66 | - |
| zma-miR156c | 228.5387 | 215.0936 | 97.2235 | -1.23 | -1.15 | - |
| zma-miR156d | 727.6565 | 585.4514 | 186.2825 | -1.97 | -1.65 | - |
| zma-miR156e | 227.4951 | 213.5613 | 96.92384 | -1.23 | -1.14 | - |
| zma-miR156f | 221.2338 | 210.7888 | 96.0999 | -1.20 | -1.13 | - |
| zma-miR156g | 221.2338 | 210.7888 | 96.0999 | -1.20 | -1.13 | - |
| zma-miR156h | 227.4951 | 213.5613 | 96.9238 | -1.23 | -1.14 | - |
| zma-miR156i | 227.4951 | 213.5613 | 96.9238 | -1.23 | -1.14 | - |
| zma-miR156k | 480.4083 | 713.6466 | 339.6079 | - | -1.07 | - |
| zma-miR156l | 227.4951 | 213.5613 | 96.9238 | -1.23 | -1.14 | - |
| zma-miR160d | 0.5963 | 0.2919 | 0.4494 | - | - | -1.03 |
| zma-miR160e | 0.5963 | 0.2919 | 0.4494 | - | - | -1.03 |
| zma-miR160g | 0.5963 | 0.2919 | 0.4494 | - | - | -1.03 |
| zma-miR164a | 997.1175 | 818.7126 | 342.8287 | -1.54 | -1.26 | - |
| zma-miR164b | 997.8629 | 819.1504 | 343.1284 | -1.54 | -1.26 | - |
| zma-miR164c | 997.7883 | 819.2234 | 343.1284 | -1.54 | -1.26 | - |
| zma-miR164d | 997.1175 | 818.7126 | 342.8287 | -1.54 | -1.26 | - |
| zma-miR164e | 1.1181 | 2.5537 | 2.5467 | 1.19 | - | 1.19 |
| zma-miR164f | 30.1886 | 24.6613 | 8.6138 | -1.81 | -1.52 | - |
| zma-miR164g | 992.6451 | 815.5752 | 341.2558 | -1.54 | -1.26 | - |
| zma-miR167a | 15.2806 | 10.0688 | 5.3930 | -1.50 | - | - |
| zma-miR167b | 15.2806 | 10.0688 | 5.3930 | -1.50 | - | - |
| zma-miR167c | 15.2806 | 10.0688 | 5.3930 | -1.50 | - | - |
| zma-miR167d | 15.2806 | 10.0688 | 5.3930 | -1.50 | - | - |
| zma-miR167e | 13.6408 | 37.8676 | 4.4192 | -1.63 | -3.10 | 1.47 |
| zma-miR167f | 13.6408 | 37.7946 | 4.4192 | -1.63 | -3.10 | 1.47 |
| zma-miR167g | 7.3794 | 3.8670 | 0.9737 | -2.92 | -1.99 | - |
| zma-miR167h | 8.3484 | 4.4507 | 1.1235 | -2.89 | -1.99 | - |
| zma-miR167i | 8.3484 | 4.4507 | 1.1235 | -2.89 | -1.99 | - |
| zma-miR167j | 13.9389 | 38.5242 | 4.4192 | -1.66 | -3.12 | 1.47 |
| zma-miR169a | 2.31073 | 1.2404 | 2.9212 | - | 1.24 | - |
| zma-miR169b | 1.63987 | 0.8026 | 2.9212 | - | 1.86 | -1.03 |
| zma-miR169c | 17.8150 | 8.4637 | 5.3181 | -1.74 | - | -1.07 |
| zma-miR169r | 17.6659 | 8.4637 | 5.3930 | -1.71 | - | -1.06 |
| zma-miR319a | 25.5671 | 10.3607 | 6.8161 | -1.91 | - | -1.30 |
| zma-miR319b | 32.9465 | 15.3951 | 11.2354 | -1.55 | - | -1.10 |
| zma-miR319c | 25.4926 | 10.3607 | 6.8161 | -1.90 | - | -1.30 |
| zma-miR319d | 32.9465 | 15.3951 | 11.2354 | -1.55 | - | -1.10 |
| zma-miR390a | 40.1024 | 88.5765 | 98.4968 | 1.30 | - | 1.14 |
| zma-miR390b | 40.1024 | 88.5765 | 98.4968 | 1.30 | - | 1.14 |
| zma-miR393a | 5.0687 | 1.0944 | 0.8988 | -2.50 | - | -2.21 |
| zma-miR393c | 5.0687 | 1.0944 | 0.8988 | -2.50 | - | -2.21 |
| zma-miR396a | 0.6709 | 1.8241 | 0.5243 | - | -1.80 | 1.44 |
| zma-miR396b | 0.6709 | 1.8241 | 0.5243 | - | -1.80 | 1.44 |
| zma-miR396c | 51.1342 | 58.0052 | 27.3394 | - | -1.09 | - |
| zma-miR396d | 51.1342 | 58.0052 | 27.3394 | - | -1.09 | - |
| zma-miR399e | 0.6709 | 1.8241 | 0.5243 | - | -1.80 | 1.44 |
| zma-miR399i | 0.6709 | 1.8241 | 0.5243 | - | -1.80 | 1.44 |
| zma-miR399j | 0.6709 | 1.8241 | 0.5243 | - | -1.80 | 1.44 |
| zma-miR528a | 15.3552 | 51.3656 | 14.0817 | - | -1.87 | 1.74 |
| zma-miR528b | 15.7279 | 52.5330 | 14.3813 | - | -1.87 | 1.74 |
| zma-miR827 | 282.7291 | 134.0322 | 100.2195 | -1.50 | - | -1.08 |

-: no significant changes.
