# Supplementary material for: Genome-Wide Identification of miRNAs and Their Targets Involved in the Developing Internodes under Maize Ears by Responding to Hormone Signaling
Source: PLoS One. 2016 Oct 3;11(10):e0164026. doi: 10.1371/journal.pone.0164026 (PMC5047619; doi:10.1371/journal.pone.0164026)
Supplement: S8 Table — (DOCX) [file pone.0164026.s009.docx]

**S8 Table**. **The conserved miRNAs showed significant changes are common to the corresponding comparison groups of the 7^th^, 8^th^ and 9^th^ internodes of ‘Xun9058’ and ‘Xun928’.**

|  | Log2 | Log2 | Log2 | Log2 | Log2 | Log2 |
| --- | --- | --- | --- | --- | --- | --- |
| miRNA | 9058-9/9058-7 | 9058-9/9058-8 | 9058-8/9058-7 | 928-9/928-7 | 928-9/928-8 | 928-8/928-7 |
| zma-miR156a | -1.54 | -1.71 | - | -1.23 | -1.15 | - |
| zma-miR156b | -1.58 | -1.72 | - | -1.97 | -1.66 | - |
| zma-miR156c | -1.54 | -1.71 | - | -1.23 | -1.15 | - |
| zma-miR156d | -1.5 | -1.62 | - | -1.97 | -1.65 | - |
| zma-miR156e | -1.52 | -1.7 | - | -1.23 | -1.14 | - |
| zma-miR156f | -1.53 | -1.7 | - | -1.2 | -1.13 | - |
| zma-miR156g | -1.53 | -1.7 | - | -1.2 | -1.13 | - |
| zma-miR156h | -1.52 | -1.7 | - | -1.23 | -1.14 | - |
| zma-miR156i | -1.52 | -1.7 | - | -1.23 | -1.14 | - |
| zma-miR156l | -1.52 | -1.7 | - | -1.23 | -1.14 | - |
| zma-miR160d | -3.27 | 1.22 | -4.49 | - | - | -1.03 |
| zma-miR160e | -3.27 | 1.22 | -4.49 | - | - | -1.03 |
| zma-miR160g | -3.27 | 1.22 | -4.49 | - | - | -1.03 |
| zma-miR164e | -1.28 | - | -2.23 | 1.19 | - | 1.19 |
| zma-miR164f | -1.1 | - | - | -1.81 | -1.52 | - |
| zma-miR164h | -1.57 | - | - | -1.54 | -1.26 | - |
| zma-miR319a | -1.57 | 2.74 | -4.31 | -1.91 | - | -1.3 |
| zma-miR319b | -1.42 | 1.95 | -3.36 | -1.55 | - | -1.1 |
| zma-miR319c | -1.57 | 2.88 | -4.45 | -1.9 | - | -1.3 |
| zma-miR319d | -1.42 | 1.95 | -3.36 | -1.55 | - | -1.1 |
| zma-miR393a | - | - | -1.67 | -2.5 | - | -2.21 |
| zma-miR393c | - | - | -1.18 | -2.5 | - | -2.21 |
| zma-miR396a | - | -1.99 | 1.96 | - | -1.8 | 1.44 |
| zma-miR396b | - | -1.99 | 1.96 | - | -1.8 | 1.44 |
| zma-miR396c | - | -1.28 | - | - | -1.09 | - |
| zma-miR396d | - | -1.28 | - | - | -1.09 | - |
| zma-miR399e | -1.47 | -1.43 | - | - | -1.8 | 1.44 |
| zma-miR399i | -1.47 | -1.43 | - | - | -1.8 | 1.44 |
| zma-miR399j | -1.47 | -1.43 | - | - | -1.8 | 1.44 |
| zma-miR528a | - | 1.07 | - | - | -1.87 | 1.74 |
| zma-miR528b | - | 1.1 | - | - | -1.87 | 1.74 |

-: no significant changes.
