# Supplementary material for: Genome-Wide Identification of miRNAs and Their Targets Involved in the Developing Internodes under Maize Ears by Responding to Hormone Signaling
Source: PLoS One. 2016 Oct 3;11(10):e0164026. doi: 10.1371/journal.pone.0164026 (PMC5047619; doi:10.1371/journal.pone.0164026)
Supplement: S9 Table — (DOCX) [file pone.0164026.s010.docx]

**S9 Table**. **The conserved miRNAs showed significant changes between the corresponding internodes of ‘Xun9058’ and ‘Xun928’.**

|  | RPM | RPM | RPM | RPM | RPM | RPM | Log2 | Log2 | Log2 |
| --- | --- | --- | --- | --- | --- | --- | --- | --- | --- |
| miRNA | 9058-7 | 9058-8 | 9058-9 | 928-7 | 928-8 | 928-9 | 9058-7/928-7 | 9058-8/928-8 | 9058-9/928-9 |
| zma-miR1432 | 3.9288 | 2.3098 | 2.9050 | 0.9690 | 1.3863 | 4.9436 | 2.02 | - | - |
| zma-miR156a | 63.6469 | 71.5348 | 21.8505 | 228.5387 | 215.0936 | 97.2234 | -1.84 | -1.59 | -2.15 |
| zma-miR156b | 124.9366 | 137.1594 | 41.7433 | 725.1967 | 581.5114 | 184.5598 | -2.54 | -2.08 | -2.14 |
| zma-miR156c | 63.6469 | 71.5348 | 21.8505 | 228.5387 | 215.0936 | 97.2234 | -1.84 | -1.59 | -2.15 |
| zma-miR156d | 127.5558 | 138.5180 | 44.9641 | 727.6565 | 585.4514 | 186.2825 | -2.51 | -2.08 | -2.05 |
| zma-miR156e | 63.1231 | 71.1951 | 21.9768 | 227.4951 | 213.5613 | 96.9238 | -1.85 | -1.58 | -2.14 |
| zma-miR156f | 62.8612 | 70.4479 | 21.7242 | 221.2338 | 210.7888 | 96.0999 | -1.82 | -1.58 | -2.15 |
| zma-miR156g | 62.8612 | 70.4479 | 21.7242 | 221.2338 | 210.7888 | 96.0999 | -1.82 | -1.58 | -2.15 |
| zma-miR156h | 63.1231 | 71.1951 | 21.9768 | 227.4951 | 213.5613 | 96.9238 | -1.85 | -1.58 | -2.14 |
| zma-miR156i | 63.1231 | 71.1951 | 21.9768 | 227.4951 | 213.5613 | 96.9238 | -1.85 | -1.58 | -2.14 |
| zma-miR156j | 19.2076 | 7.8804 | 17.9983 | 6.7086 | 7.3692 | 13.2577 | 1.52 | - | - |
| zma-miR156k | 335.8708 | 304.4815 | 182.2560 | 480.4083 | 713.6466 | 339.6079 | - | -1.23 | - |
| zma-miR156l | 63.1231 | 71.1951 | 21.9768 | 227.4951 | 213.5613 | 96.9238 | -1.85 | -1.58 | -2.14 |
| zma-miR159a | 354.8164 | 536.6810 | 194.4443 | 480.4083 | 403.3369 | 507.9139 | - | - | -1.39 |
| zma-miR159b | 354.3799 | 535.1185 | 194.0023 | 479.3648 | 402.6073 | 507.0150 | - | - | -1.39 |
| zma-miR159f | 354.8164 | 536.6810 | 194.3180 | 480.3338 | 403.1910 | 507.8390 | - | - | -1.39 |
| zma-miR159j | 354.3799 | 535.0506 | 193.8760 | 479.2902 | 402.4613 | 506.9401 | - | - | -1.39 |
| zma-miR159k | 354.3799 | 535.0506 | 193.8760 | 479.2902 | 402.4613 | 506.9401 | - | - | -1.39 |
| zma-miR160a | 3.0558 | 0.1359 | 0.2526 | 0.5218 | 0.2919 | 0.3745 | 2.55 | -1.10 | - |
| zma-miR160b | 3.0558 | 0.1359 | 0.2526 | 0.5218 | 0.2919 | 0.3745 | 2.55 | -1.10 | - |
| zma-miR160c | 3.0558 | 0.1359 | 0.2526 | 0.5218 | 0.2919 | 0.3745 | 2.55 | -1.10 | - |
| zma-miR160d | 3.0558 | 0.1359 | 0.3158 | 0.5963 | 0.2919 | 0.4494 | 2.36 | -1.10 | - |
| zma-miR160e | 3.0558 | 0.1359 | 0.3158 | 0.5963 | 0.2919 | 0.4494 | 2.36 | -1.10 | - |
| zma-miR160g | 3.0558 | 0.1359 | 0.3158 | 0.5963 | 0.2919 | 0.4494 | 2.36 | -1.10 | - |
| zma-miR162 | 18.6837 | 14.0624 | 9.9780 | 9.0193 | 9.7770 | 7.1906 | 1.05 | - | - |
| zma-miR164a | 319.1951 | 192.1861 | 183.1402 | 997.1175 | 818.7126 | 342.8287 | -1.64 | -2.09 | - |
| zma-miR164b | 319.6316 | 192.5258 | 183.5822 | 997.8629 | 819.1504 | 343.1284 | -1.64 | -2.09 | - |
| zma-miR164c | 319.7189 | 192.6617 | 183.7085 | 997.7883 | 819.2234 | 343.1284 | -1.64 | -2.09 | - |
| zma-miR164d | 319.1951 | 192.1861 | 183.1402 | 997.1175 | 818.7126 | 342.8287 | -1.64 | -2.09 | - |
| zma-miR164e | 4.4527 | 0.9511 | 1.8314 | 1.1181 | 2.5537 | 2.5467 | 1.99 | -1.42 | - |
| zma-miR164f | 13.4453 | 9.9184 | 6.2520 | 30.1886 | 24.6613 | 8.6138 | -1.17 | -1.31 | - |
| zma-miR164g | 304.2656 | 184.1020 | 175.4356 | 992.6451 | 815.5752 | 341.2558 | -1.71 | -2.15 | - |
| zma-miR167a | 3.4923 | 3.5326 | 2.2103 | 15.2806 | 10.0688 | 5.3930 | -2.13 | -1.51 | -1.29 |
| zma-miR167b | 3.4923 | 3.5326 | 2.2735 | 15.2806 | 10.0688 | 5.3930 | -2.13 | -1.51 | -1.25 |
| zma-miR167c | 3.4923 | 3.5326 | 2.2103 | 15.2806 | 10.0688 | 5.3930 | -2.13 | -1.51 | -1.29 |
| zma-miR167d | 3.4923 | 3.5326 | 2.2103 | 15.2806 | 10.0688 | 5.3930 | -2.13 | -1.51 | -1.29 |
| zma-miR167e | 2.8811 | 3.8723 | 2.5261 | 13.6408 | 37.8676 | 4.4192 | -2.24 | -3.29 | - |
| zma-miR167f | 2.8811 | 3.8723 | 2.5261 | 13.6408 | 37.7946 | 4.4192 | -2.24 | -3.29 | - |
| zma-miR167g | 1.9208 | 2.2418 | 1.7051 | 7.3794 | 3.8670 | 0.9737 | -1.94 | - | - |
| zma-miR167h | 2.0954 | 2.5815 | 1.8946 | 8.3484 | 4.4507 | 1.1235 | -1.99 | - | - |
| zma-miR167i | 2.0954 | 2.5815 | 1.8946 | 8.3484 | 4.4507 | 1.1235 | -1.99 | - | - |
| zma-miR167j | 2.8811 | 3.8723 | 2.5892 | 13.9389 | 38.5242 | 4.4192 | -2.27 | -3.31 | - |
| zma-miR169a | 2.5319 | 3.3288 | 4.1680 | 2.3107 | 1.2404 | 2.9212 | - | 1.42 | - |
| zma-miR169b | 2.6192 | 3.1929 | 4.1049 | 1.6399 | 0.8026 | 2.9212 | - | 1.99 | - |
| zma-miR169r | 2.7065 | 2.8532 | 1.6419 | 17.6659 | 8.4637 | 5.3930 | -2.71 | -1.57 | -1.72 |
| zma-miR171d | 1.2223 | 1.6304 | 1.4525 | 2.4598 | 2.0430 | 1.4981 | -1.01 | - | - |
| zma-miR171e | 1.2223 | 1.6304 | 1.4525 | 2.4598 | 2.0430 | 1.4981 | -1.01 | - | - |
| zma-miR171f | 1.2223 | 1.6304 | 1.5788 | 2.5343 | 1.9700 | 1.5730 | -1.05 | - | - |
| zma-miR171h | 1.1350 | 0.5435 | 1.3893 | 0.6709 | 1.2404 | 1.1235 | - | -1.19 | - |
| zma-miR171i | 1.2223 | 1.6304 | 1.5788 | 2.5343 | 1.9700 | 1.5730 | -1.05 | - | - |
| zma-miR171k | 1.1350 | 0.5435 | 1.3893 | 0.6709 | 1.2404 | 1.1235 | - | -1.19 | - |
| zma-miR171l | 0.1746 | 0.5435 | 0.4421 | 0.4472 | 0.5107 | 0.2996 | -1.36 | - | - |
| zma-miR171m | 0.1746 | 0.5435 | 0.4421 | 0.4472 | 0.5107 | 0.2996 | -1.36 | - | - |
| zma-miR172a | 344.3396 | 673.6365 | 575.0601 | 229.4332 | 138.1181 | 245.6053 | - | 2.29 | 1.23 |
| zma-miR172b | 344.4269 | 673.7045 | 575.0601 | 229.4332 | 138.1181 | 245.6053 | - | 2.29 | 1.23 |
| zma-miR172c | 344.4269 | 673.7045 | 575.0601 | 229.4332 | 138.1181 | 245.6053 | - | 2.29 | 1.23 |
| zma-miR172d | 344.4269 | 673.7045 | 575.0601 | 229.4332 | 138.1181 | 245.6053 | - | 2.29 | 1.23 |
| zma-miR319a | 29.6844 | 1.4946 | 9.9780 | 25.5671 | 10.3607 | 6.8161 | - | -2.79 | - |
| zma-miR319b | 33.5260 | 3.2608 | 12.5672 | 32.9465 | 15.3951 | 11.2354 | - | -2.24 | - |
| zma-miR319c | 29.5971 | 1.3587 | 9.9780 | 25.4926 | 10.3607 | 6.8161 | - | -2.93 | - |
| zma-miR319d | 33.5260 | 3.2608 | 12.5672 | 32.9465 | 15.3951 | 11.2354 | - | -2.24 | - |
| zma-miR393a | 7.7703 | 2.4456 | 4.6101 | 5.0687 | 1.0944 | 0.8988 | - | 1.16 | 2.36 |
| zma-miR396a | 1.4842 | 5.7744 | 1.4525 | 0.6709 | 1.8241 | 0.5243 | 1.15 | 1.66 | 1.47 |
| zma-miR396b | 1.4842 | 5.7744 | 1.4525 | 0.6709 | 1.8241 | 0.5243 | 1.15 | 1.66 | 1.47 |
| zma-miR396c | 20.9537 | 27.1058 | 11.1779 | 51.1342 | 58.0052 | 27.3394 | -1.29 | -1.10 | -1.29 |
| zma-miR396d | 20.9537 | 27.1058 | 11.1779 | 51.1342 | 58.0052 | 27.3394 | -1.29 | -1.10 | -1.29 |
| zma-miR399e | 0.3492 | 0.3397 | 0.1263 | 0.6709 | 1.8241 | 0.5243 | - | -2.42 | -2.05 |
| zma-miR399i | 0.3492 | 0.3397 | 0.1263 | 0.6709 | 1.8241 | 0.5243 | - | -2.42 | -2.05 |
| zma-miR399j | 0.3492 | 0.3397 | 0.1263 | 0.6709 | 1.8241 | 0.5243 | - | -2.42 | -2.05 |
| zma-miR528a | 22.4379 | 15.8287 | 33.3441 | 15.3552 | 51.3656 | 14.0817 | - | -1.70 | 1.24 |
| zma-miR528b | 25.0572 | 16.8477 | 36.0597 | 15.7279 | 52.5330 | 14.3813 | - | -1.64 | 1.33 |

-: no significant changes.
