# Supplementary material for: Genome-Wide Identification of miRNAs and Their Targets Involved in the Developing Internodes under Maize Ears by Responding to Hormone Signaling
Source: PLoS One. 2016 Oct 3;11(10):e0164026. doi: 10.1371/journal.pone.0164026 (PMC5047619; doi:10.1371/journal.pone.0164026)
Supplement: S10 Table — (DOCX) [file pone.0164026.s011.docx]

**S10 Table**. **The conserved miRNAs showed significant changes at least in two internodes of ‘Xun928’ compared with the corresponding internodes of ‘Xun9058’.**

|  | Log2 | Log2 | Log2 |
| --- | --- | --- | --- |
| miRNA | 9058-7/928-7 | 9058-8/928-8 | 9058-9/928-9 |
| zma-miR156a | -1.84 | -1.59 | -2.15 |
| zma-miR156b | -2.54 | -2.08 | -2.14 |
| zma-miR156c | -1.84 | -1.59 | -2.15 |
| zma-miR156d | -2.51 | -2.08 | -2.05 |
| zma-miR156e | -1.85 | -1.58 | -2.14 |
| zma-miR156f | -1.82 | -1.58 | -2.15 |
| zma-miR156g | -1.82 | -1.58 | -2.15 |
| zma-miR156h | -1.85 | -1.58 | -2.14 |
| zma-miR156i | -1.85 | -1.58 | -2.14 |
| zma-miR156l | -1.85 | -1.58 | -2.14 |
| zma-miR160a | 2.55 | -1.1 | - |
| zma-miR160b | 2.55 | -1.1 | - |
| zma-miR160c | 2.55 | -1.1 | - |
| zma-miR160d | 2.36 | -1.1 | - |
| zma-miR160e | 2.36 | -1.1 | - |
| zma-miR160g | 2.36 | -1.1 | - |
| zma-miR164a | -1.64 | -2.09 | - |
| zma-miR164b | -1.64 | -2.09 | - |
| zma-miR164c | -1.64 | -2.09 | - |
| zma-miR164d | -1.64 | -2.09 | - |
| zma-miR164e | 1.99 | -1.42 | - |
| zma-miR164f | -1.17 | -1.31 | - |
| zma-miR164g | -1.71 | -2.15 | - |
| zma-miR167a | -2.13 | -1.51 | -1.29 |
| zma-miR167b | -2.13 | -1.51 | -1.25 |
| zma-miR167c | -2.13 | -1.51 | -1.29 |
| zma-miR167d | -2.13 | -1.51 | -1.29 |
| zma-miR167e | -2.24 | -3.29 | - |
| zma-miR167f | -2.24 | -3.29 | - |
| zma-miR167j | -2.27 | -3.31 | - |
| zma-miR169r | -2.71 | -1.57 | -1.72 |
| zma-miR172a | - | 2.29 | 1.23 |
| zma-miR172b | - | 2.29 | 1.23 |
| zma-miR172c | - | 2.29 | 1.23 |
| zma-miR172d | - | 2.29 | 1.23 |
| zma-miR393a | - | 1.16 | 2.36 |
| zma-miR396a | 1.15 | 1.66 | 1.47 |
| zma-miR396b | 1.15 | 1.66 | 1.47 |
| zma-miR396c | -1.29 | -1.1 | -1.29 |
| zma-miR396d | -1.29 | -1.1 | -1.29 |
| zma-miR399e | - | -2.42 | -2.05 |
| zma-miR399i | - | -2.42 | -2.05 |
| zma-miR399j | - | -2.42 | -2.05 |
| zma-miR528a | - | -1.7 | 1.24 |
| zma-miR528b | - | -1.64 | 1.33 |

-: no significant changes.
