# Supplementary material for: Genome-Wide Identification of miRNAs and Their Targets Involved in the Developing Internodes under Maize Ears by Responding to Hormone Signaling
Source: PLoS One. 2016 Oct 3;11(10):e0164026. doi: 10.1371/journal.pone.0164026 (PMC5047619; doi:10.1371/journal.pone.0164026)
Supplement: S11 Table — (DOCX) [file pone.0164026.s012.docx]

**S11 Table. Novel miRNAs co-detected in six internodes libraries.**

| Name | Chromosome | Location | Arm | Precursor  L(nt) | Free energy  (kcal/mol) | Mature sequence(5'-3') | L(nt) | MFEIs |
| --- | --- | --- | --- | --- | --- | --- | --- | --- |
| zma-miRn1 | chr8 | 4791964:4792141:+ | 5p | 178 | -98.5 | TTAGGCTCGGGGACTACGGTG | 21 | 1.036857 |
| zma-miRn2a | chr1 | 240998450:240998591:- | 3p | 142 | -53.4 | TTGGATTTTGATTGGATGCAC | 21 | 0.95349 |
| zma-miRn2b | chr4 | 156412142:156412315:+ | 3p | 174 | -73.8 | TTGGATTTTGATTGGATGCAC | 21 | 1.190396 |
| zma-miRn3 | chr1 | 245903450:245903553:+ | 3p | 104 | -66.3 | TTGAGCCGCGTCAATATCTCC | 21 | 1.086957 |
