# Supplementary material for: Genome-Wide Identification of miRNAs and Their Targets Involved in the Developing Internodes under Maize Ears by Responding to Hormone Signaling
Source: PLoS One. 2016 Oct 3;11(10):e0164026. doi: 10.1371/journal.pone.0164026 (PMC5047619; doi:10.1371/journal.pone.0164026)
Supplement: S12 Table — (DOCX) [file pone.0164026.s013.docx]

**S12 Table. The expression changes of novel miRNAs for each pairwise comparison among the 7^th^, 8^th^ and 9^th^ internodes of ‘Xun9058’.**

|  | RPM | RPM | RPM | Log2 | Log2 | Log2 |
| --- | --- | --- | --- | --- | --- | --- |
| Name | 9058-7 | 9058-8 | 9058-9 | 9058-9/9058-7 | 9058-9/9058-8 | 9058-8/9058-7 |
| zma-miRn1 | 264.2789 | 322.4841 | 358.1969 | - | - | - |
| zma-miRn2a | 9.8657 | 9.4429 | 8.6518 | - | - | - |
| zma-miRn2b | 9.7784 | 9.4429 | 8.4623 | - | - | - |
| zma-miRn3 | 8.2942 | 11.8885 | 9.6622 | - | - | - |

-: no significant changes.
