# Supplementary material for: Genome-Wide Identification of miRNAs and Their Targets Involved in the Developing Internodes under Maize Ears by Responding to Hormone Signaling
Source: PLoS One. 2016 Oct 3;11(10):e0164026. doi: 10.1371/journal.pone.0164026 (PMC5047619; doi:10.1371/journal.pone.0164026)
Supplement: S13 Table — (DOCX) [file pone.0164026.s014.docx]

**S13 Table.** **The expression changes of novel miRNAs for each pairwise comparison among the 7^th^, 8^th^ and 9^th^ internodes of ‘Xun928’.**

|  | RPM | RPM | RPM | Log2 | Log2 | Log2 |
| --- | --- | --- | --- | --- | --- | --- |
| Name | 928-7 | 928-8 | 928-9 | 928-9/928-7 | 928-9/928-8 | 928-8/928-7 |
| zma-miRn1 | 118.8163 | 119.7315 | 183.4362 | - | - | - |
| zma-miRn2a | 9.1684 | 12.7684 | 9.8871 | - | - | - |
| zma-miRn2b | 8.9448 | 12.5496 | 9.8871 | - | - | - |
| zma-miRn3 | 11.9264 | 15.9058 | 16.3288 | - | - | - |

-: no significant changes.
