# Supplementary material for: Genome-Wide Identification of miRNAs and Their Targets Involved in the Developing Internodes under Maize Ears by Responding to Hormone Signaling
Source: PLoS One. 2016 Oct 3;11(10):e0164026. doi: 10.1371/journal.pone.0164026 (PMC5047619; doi:10.1371/journal.pone.0164026)
Supplement: S14 Table — (DOCX) [file pone.0164026.s015.docx]

**S14 Table. The expression changes of novel miRNAs between the corresponding internodes of ‘Xun9058’ and ‘Xun928’.**

|  | Log2 | Log2 | Log2 |
| --- | --- | --- | --- |
| Name | 9058-7/928-7 | 9058-8/928-8 | 9058-9/928-9 |
| zma-miRn1 | 1.15 | 1.43 | - |
| zma-miRn2a | - | - | - |
| zma-miRn2b | - | - | - |
| zma-miRn3 | - | - | - |

-: no significant changes.
