# Supplementary material for: Genome-Wide Identification of miRNAs and Their Targets Involved in the Developing Internodes under Maize Ears by Responding to Hormone Signaling
Source: PLoS One. 2016 Oct 3;11(10):e0164026. doi: 10.1371/journal.pone.0164026 (PMC5047619; doi:10.1371/journal.pone.0164026)
Supplement: S15 Table — (DOCX) [file pone.0164026.s016.docx]

**S15 Table. Internode development associated with maize internode miRNA target genes detected via a genome wide degradome.**

| SmallRNA | Target | AlignmentScore | AlignmentRange | CleavageSite | GO |
| --- | --- | --- | --- | --- | --- |
| zma-miR156a-I,l | GRMZM2G148467_T02 | 1 | 984-1003 | 994 | Unknown |
|  | GRMZM2G148467_T01 | 1 | 836-855 | 846 | SPL protein 10 |
|  | GRMZM2G097275_T04 | 1 | 1360-1379 | 1370 | SPL protein 2 |
|  | GRMZM2G097275_T02 | 1 | 2063-2082 | 2073 | SPL protein 2 |
|  | GRMZM2G097275_T01 | 1 | 2063-2082 | 2073 | SPL protein 2 |
|  | GRMZM2G097275_T03 | 1 | 2063-2082 | 2073 | SPL protein 2 |
|  | GRMZM2G016439_T02 | 4 | 332-351 | 342 | histidine-containing phosphotransfer factor 5 |
|  | GRMZM2G016439_T01 | 4 | 351-370 | 361 | histidine-containing phosphotransfer factor 5 |
| zma-miR160a-e,g | GRMZM2G159399_T01 | 1 | 1785-1805 | 1796 | auxin response factor 16 |
| zma-miR164a | GRMZM2G040561_T02 | 4 | 1958-1979 | 1969 | Protein binding |
| zma-miR164a-d,g | GRMZM2G063522_T01 | 2 | 895-915 | 906 | NAC domain protein NAC1 |
| zma-miR164f | GRMZM2G063522_T01 | 1 | 895-915 | 906 | NAC domain protein NAC1 |
| zma-miR164h | GRMZM2G063522_T01 | 2.5 | 895-915 | 906 | NAC domain protein NAC1 |
| zma-miR167a-d | GRMZM2G475882_T02 | 4 | 2648-2669 | 2659 | auxin response factor 6 |
|  | GRMZM2G078274_T03 | 4 | 2500-2521 | 2511 | auxin response factor 6 |
|  | GRMZM2G078274_T02 | 4 | 187-208 | 198 | auxin response factor 8 |
|  | GRMZM2G078274_T01 | 4 | 2500-2521 | 2511 | auxin response factor 6 |
|  | GRMZM2G475882_T01 | 4 | 2530-2551 | 2541 | auxin response factor 6 |
| zma-miR167e-j | GRMZM2G110881_T03 | 4 | 838-855 | 846 | NAD(P)-binding Rossmann-fold superfamily protein |
|  | GRMZM2G110881_T01 | 4 | 798-815 | 806 | NAD(P)-binding Rossmann-fold superfamily protein |
| zma-miR169r | GRMZM2G037630_T01 | 3.5 | 796-816 | 807 | NF-YA6 |
|  | GRMZM2G040349_T01 | 3.5 | 2144-2164 | 2155 | NF-YA3 |
|  | GRMZM2G040349_T02 | 3.5 | 1873-1893 | 1884 | NF-YA3 |
|  | GRMZM2G000686_T06 | 2.5 | 890-910 | 901 | NF-YA6 |
|  | GRMZM2G000686_T10 | 2.5 | 1560-1580 | 1571 | NF-YA3 |
|  | GRMZM2G000686_T01 | 2.5 | 2120-2140 | 2131 | NF-YA3 |
|  | GRMZM2G000686_T04 | 2.5 | 962-982 | 973 | NF-YA3 |
|  | GRMZM2G000686_T05 | 2.5 | 1765-1785 | 1776 | NF-YA6 |
|  | GRMZM2G000686_T03 | 2.5 | 1837-1857 | 1848 | NF-YA3 |
|  | GRMZM2G000686_T08 | 2.5 | 1543-1563 | 1554 | NF-YA5 |
|  | GRMZM2G000686_T02 | 2.5 | 1816-1836 | 1827 | NF-YA3 |
| zma-miR172a-d | GRMZM6G798998_T01 | 4 | 1007-1027 | 1018 | ATP binding |
|  | GRMZM2G057525_T01 | 3.5 | 1387-1405 | 1396 | Unknown |
| zma-miR319a-d | GRMZM2G412073_T01 | 3.5 | 232-251 | 242 | Unknown |
|  | GRMZM2G020805_T01 | 3 | 1429-1448 | 1439 | TCP family transcription factor 2 |
|  | GRMZM2G089361_T01 | 2.5 | 1316-1335 | 1326 | TCP family transcription factor 2 |
|  | GRMZM2G015037_T02 | 4 | 1594-1613 | 1604 | TCP family transcription factor 4 |
|  | GRMZM2G015037_T01 | 4 | 1735-1754 | 1745 | TCP family transcription factor 4 |
|  | GRMZM2G028054_T02 | 2 | 1693-1712 | 1703 | myb domain protein 65 |
|  | GRMZM2G028054_T03 | 2 | 1693-1712 | 1703 | myb domain protein 65 |
|  | GRMZM2G028054_T01 | 2 | 1693-1712 | 1703 | myb domain protein 65 |
|  | GRMZM2G103825_T01 | 2.5 | 822-840 | 831 | fatty acid amide hydrolase |
| zma-miR393a-c | GRMZM5G848945_T02 | 1 | 1737-1757 | 1748 | auxin signaling F-BOX 2 |
|  | GRMZM2G137451_T02 | 3.5 | 1973-1994 | 1985 | auxin signaling F-BOX 2 |
|  | GRMZM2G137451_T01 | 3.5 | 1678-1699 | 1690 | auxin signaling F-BOX 2 |
| zma-miR396a,b | GRMZM2G033612_T02 | 3 | 1008-1029 | 1019 | growth-regulating factor 2 |
|  | GRMZM2G041223_T01 | 3 | 887-908 | 898 | growth-regulating factor 5 |
|  | GRMZM2G034876_T01 | 3 | 662-683 | 673 | growth-regulating factor 5 |
|  | GRMZM2G034876_T03 | 3 | 662-683 | 673 | growth-regulating factor 5 |
|  | GRMZM2G034876_T02 | 3 | 662-683 | 673 | growth-regulating factor 5 |
|  | GRMZM2G024293_T03 | 4 | 1035-1054 | 1045 | nucleotide binding |
|  | GRMZM2G024293_T01 | 4 | 1181-1200 | 1191 | nucleotide binding |
|  | GRMZM2G045977_T01 | 3 | 801-822 | 812 | growth-regulating factor 3 |
|  | GRMZM2G099862_T02 | 3 | 557-578 | 568 | growth-regulating factor 2 |
|  | GRMZM2G099862_T04 | 3 | 444-465 | 455 | growth-regulating factor 2 |
|  | GRMZM2G099862_T03 | 3 | 643-664 | 654 | growth-regulating factor 2 |
|  | GRMZM2G099862_T01 | 3 | 690-711 | 701 | growth-regulating factor 2 |
|  | GRMZM2G129147_T01 | 3 | 639-660 | 650 | growth-regulating factor 5 |
|  | GRMZM2G129147_T02 | 3 | 819-840 | 830 | Unknown |
| zma-miR396c,d | GRMZM2G129147_T01 | 1 | 639-660 | 651 | growth-regulating factor 5 |
|  | GRMZM2G129147_T02 | 1 | 819-840 | 831 | Unknown |
|  | GRMZM2G041223_T01 | 1 | 887-908 | 899 | growth-regulating factor 5 |
| zma-miR399e,I,j | GRMZM2G002807_T01 | 4 | 635-654 | 645 | triose-phosphate isomerase activity |
